# Supplementary material for: Causal Relationship between Aging and Anorexia Nervosa: A White-Matter-Microstructure-Mediated Mendelian Randomization Analysis
Source: Biomedicines. 2024 Aug 16;12(8):1874. doi: 10.3390/biomedicines12081874 (PMC11351342; doi:10.3390/biomedicines12081874)
Supplement: Supplementary file 1 [file biomedicines-12-01874-s001.zip › Supplementary Figure/Figure S9/Leaveoneout plot of MR analysis for ubm-b-1483 to AN.pdf]

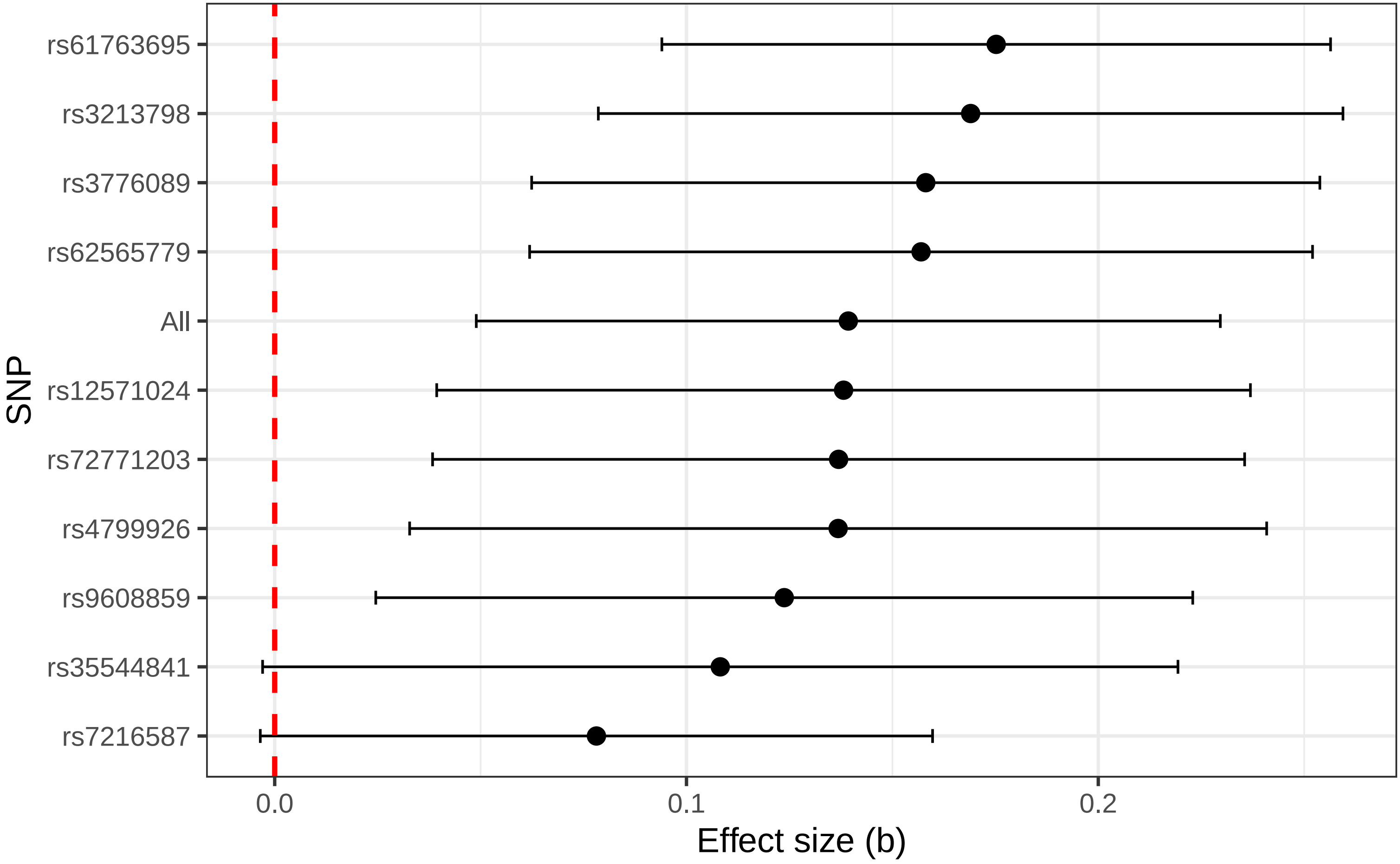

rs61763695
rs3213798
rs3776089
rs62565779
All
rs12571024
rs72771203
rs4799926
rs9608859
rs35544841
rs7216587
SNP
0.0
0.1
0.2
Effect size (b)
